# Cell studio: A platform for interactive, 3D graphical simulation of immunological processes

**DOI:** 10.1063/1.5039473

**Published:** 2018-06-04

**Authors:** Asaf Liberman, Danny Kario, Matan Mussel, Jacob Brill, Kenneth Buetow, Sol Efroni, Uri Nevo

**Affiliations:** 1 The Iby and Aladar Fleischman Faculty of Engineering, Tel Aviv University, Tel Aviv 6997801, Israel; 2 Sagol School of Neuroscience, Tel Aviv University, Tel Aviv 6997801, Israel; 3 Physics Department, TU Dortmund University, Dortmund 44227, Germany; 4 Arizona State University, Tempe, Arizona 85281, USA; 5 The Mina and Everard Goodman Faculty of Life Sciences, Bar Ilan University, Ramat Gan 52900, Israel

## Abstract

The field of computer modeling and simulation of biological systems is rapidly advancing, backed by significant progress in the fields of experimentation techniques, computer hardware, and programming software. The result of a simulation may be delivered in several ways, from numerical results, through graphs of the simulated run, to a visualization of the simulation. The vision of an *in-silico* experiment mimicking an *in-vitro* or *in-vivo* experiment as it is viewed under a microscope is appealing but technically demanding and computationally intensive. Here, we report “Cell Studio,” a generic, hybrid platform to simulate an immune microenvironment with biological and biophysical rules. We use game engines—generic programs for game creation which offer ready-made assets and tools—to create a visualized, interactive 3D simulation. We also utilize a scalable architecture that delegates the computational load to a server. The user may view the simulation, move the “camera” around, stop, fast-forward, and rewind it and inject soluble molecules into the extracellular medium at any point in time. During simulation, graphs are created in real time for a broad view of system-wide processes. The model is parametrized using a user-friendly Graphical User Interface (GUI). We show a simple validation simulation and compare its results with those from a “classical” simulation, validated against a “wet” experiment. We believe that interactive, real-time 3D visualization may aid in generating insights from the model and encourage intuition about the immunological scenario.

## INTRODUCTION

Computer models aim to incorporate multi-scale biological data into comprehensive, dynamic computer software that simulates a biological system. Such models may help in understanding and even predicting the outcome of the modeled biological processes.^1–4^ Advance in computer modeling is fueled by both advance in biological experimentation techniques (the so-called “-omics”)^5^—which significantly increase the amount of data acquired in a given experiment—and advance in computer hardware technology—which boosts the resources available to the modeler. Biological models are at the core of Systems Biology, a novel approach to analyzing biological systems as a dynamic whole, which may overcome some of the limitations posed by more reductionist approaches.^2,6–8^

## APPROACHES TO BIOLOGICAL MODELING

Biological modeling paradigms can be roughly associated with one of the two main categories: mathematical modeling and computational modeling. Mathematical models use mathematical tools, most commonly differential equations, to calculate dynamic relationships between bulk quantities.^9,10^ In contrast, computational models use computer algorithms to describe a system, frequently depicting each entity separately.^6,9^

Such is Agent-Based Modeling (ABM), a modeling paradigm which attempts to break down a system into its comprising entities, treating each of them as a separate agent, capable of making its own “decisions,” based on its local environment.^11,12^ Biological models may concentrate on different biological scales, from single molecules, through the cellular scale, to the entire organism. “Middle–out” biological simulations, which introduce the cell as the basic unit of function, are naturally well suited to ABM.^5,10^ This paradigm enables the assignment of known cellular characteristics to each cell-agent, without the need to assert the systemic impact of cell-cell and cell-lattice interactions. Thus, data obtained from biological experiments, which are otherwise hard to grasp due to their size and level of detail, are incorporated into a model and simulated to be analyzed at the system level. The discrete nature of the simulation makes it possible to track individual cells and follow populations, to observe how mesoscopic entities' actions and interactions lead to macro-level events—a phenomenon referred to as emergent behavior.^9,11,13^ A middle-out, cellular-level model may later be extended “down” and “up”—either by adding intra-cellular mechanisms (e.g., signaling pathways) or by adding macroscopic phenomena, at the organ- or entire body-level, respectively, thus creating a multiscale model.^5^

## CURRENTLY AVAILABLE FRAMEWORKS

In recent years, several teams have built general purpose simulation systems, software frameworks that offer basic tools that should serve as building blocks to allow researchers to create an *ad-hoc* simulation for a particular biological case. These simulation systems can be classified, apart from their aforementioned modeling paradigm, by their modelled biological scale: from the intracellular level to the complete organism level. Intracellular scale simulations model different phenomena occurring inside a cell or on its surface, concentrating on a single cell per simulation run. Standout examples of intracellular, general purpose systems are Virtual Cell,^14,15^ BIOCHAM,^16^ BioNetGen,^17^ Cellular Dynamic Simulator (CDS),^18^ Smoldyn,^19,20^ and COPASI,^21^ which simulate different intracellular processes using different modeling paradigms. Some systems listed above may be used either standalone or incorporated into other systems (e.g., Virtual Cell incorporates Smoldyn and COPASI) to serve as an algorithmic solution for a certain component in the process. For further information, we refer to the reader to reviews of such systems.^22,23^

Cellular level simulation systems model the mesoscopic scale—cells and extracellular matrix, often along with the extracellular fluid and soluble molecules. These systems commonly adopt the ABM paradigm for its natural fit to modelling cells. Using ABM facilitates both the development of the modeling system—since Object Oriented Programming, the software architecture equivalent to agent-based modeling, is a prominent software engineering pattern—and the researcher's work of creating the model—since assigning properties and abilities to each cell population separately emulates the biological study process more closely. In addition, this modeling method may alleviate the need for advanced mathematical or algorithmic knowledge required to create a model.^24–27^

Examples of cellular level, agent based, general purpose simulation systems include the Multiscale Systems Immunology Project (MSI),^28^ EPISIM,^29,30^ LINDSAY,^31^ SimuLife,^32^ FLAME,^33–35^ Simmune,^36,37^ C-ImmSim,^38^ and CellSys.^39^ Also mentionable is Compucell3D,^40,41^ a prominent, non-agent-based, biological simulation system. See Table I for a concise comparison of these systems. There are also projects with the stated strategy of combining several simulation systems into one framework, using a shared specification, such as Multiscale Modelling and Simulation Framework (MMSF)^42^ and Fully integrated Immune Response Model (FIRM).^43^ Reviewing these systems in depth is outside the scope of this article, and we refer the reader to the individual articles referenced or to detailed reviews of these systems in Refs. 13 and 44.

**TABLE I.** A comparison of frameworks for biological modeling and simulation that include modeling of the cellular level. It should be noted that newer versions of the surveyed software may contain features that are not mentioned in this table.

Name of systems	Biological scale	Paradigm	Medium	Visualization capabilities	Real-time interactivity	Features for scalability
MSI^28^	Multiscale-cellular and intracellular	Agent-based	Yes	Non-real-time 3D	None	Parallelization
EPISIM^29,30^	Multiscale-cellular and intracellular using COPASI	Multi paradigm	No	Real-time 2D and 3D	Simulation parameter control	Parallelization
LINDSAY^31^	Cellular	Agent-based	No	Real-time 3D visualization	Moving camera, simulation parameter control	Distributed computing
SimuLife^32^	Cellular	“Reactive animation”	Yes	Real-time 3D	Moving camera, simulation parameter control	Client–server architecture on web
FLAME-GPU^33–35^	Cellular, subcellular if linked to COPASI	Agent-based	No, but can be linked to other simulators to enable a medium	3D visualization	In later versions	GPU used for non-graphical tasks
Simmune^36,37^	Multiscale	Multi-paradigm–agent based, 3D grid	Yes	Non-graphical output of data	No	No
C-ImmSim^38^	Multiscale–cellular and intracellular	Agent-based	Yes	Non-graphical output of data	No	No, but following system “ImmunoGrid” uses grid technologies for scalability^45^
CellSys^39^	Cellular	Agent-based	Yes	Real-time 3D with medium field visualization	No	Parallelization using OpenMP
Compucell3D^40^	Cellular	Cellular Potts model (CPM) or lattice-based Glazier–Graner–Hogeweg (GGH)	Yes	Real-time 3D	No	Parallelization using OpenMP

As can be seen in Table I, these systems differ in several key aspects, among them: simulation scale, modeling paradigm (using a single modeling approach or a “hybrid” of several paradigms), inclusion of the extracellular medium along with biophysics of soluble molecules, their visualization capabilities (real-time or otherwise), the level of in-simulation interactivity, and their use of different algorithmic measures for scalability and performance enhancement. These differences were central in our decision to build a new system which attempts to combine some of these systems' abilities while emphasizing the important aspect of real-time, 3D, interactive visualization of a simulation, and its scalability, using game engine technology.

## COMPUTER MODELING TOOLS

The video game industry has seen a steady growth in recent years—particularly thanks to a surge in mobile gaming^46^—and with it, the need for high quality game engines. Game engines are programs that provide tools and assets that facilitate and expedite game development, which include in part: a scripting environment, network capabilities, a physics engine, a rendering engine, and an extensive IDE (Integrated Development Environment) for centralized control of the development process. Using game engines for uses other than game creation (e.g., architecture, medical, etc.) is referred to as “serious games.”^47,48^

A great challenge of modeling the immune system is dealing with the vast number and a large variety of entities to model, while running the simulation on a single Central Processing Unit (CPU) computer, a standard desktop client. There are several approaches for pushing the computational power beyond what is made available in a single processor: (a) repurposing the GPU for parallelization of trivial non-graphical calculation^35,49^ (the GPU is built for tasks of different nature from CPU, making it better equipped to handle many simple calculations in parallel), (b) parallelizing the simulation over several threads, using multicore CPUs,^18,31,42,50^ and (c) using a client-server architecture, delegating heavy duty calculations to the “server” while assigning simpler, mainly visualization-oriented tasks to the client,^32^ thereby removing the requirement for an expensive, high performance computer from the end-user.

In this work, we describe “Cell Studio” (available for download at www.cellstudio.info, currently only for the Windows OS), a general-purpose, multi-paradigm (“hybrid”) framework to simulate and visualize an immune microenvironment in real-time 3D, while allowing interaction with the simulation during the run [moving the camera freely, controlling the timeline (including to rewind the simulation), injecting soluble molecules into the medium, etc.]. We deploy a client-server architecture, using the Unity3D game engine at the client side and a scalable C++ algorithm at the server side, using the GPU to accelerate certain (non-graphical) processes. We offer an intuitive graphical user interface (GUI) for parameter initialization at the beginning of the modeling process. A “batch mode” is available, enabling the user to run many simulations continuously with a textual output instead of visualization.

We refer here to a hybrid model in two functions: (a) it combines discrete and continuous forms of calculation^10^ and (b) it combines an agent-based simulation of independent simulation entities (e.g., cells) with functional programming for describing the medium (e.g., soluble molecule diffusion).

Combining the concept of agent based modelling with strong visualization capabilities is known to assist biologists (and other “domain experts”) with little formal training in advanced mathematical or computer algorithms to use a simulation system.^15,24,27,31,32^ Our main goal in creating this framework is to use the tools offered by game engines to overcome challenges in interactive, real-time 3D visualization and create a system that is user-friendly and approachable.

## RESULTS

### Parameter initialization

Each cell is an agent in the simulation and has properties that are saved in its object class. A cell object is not characterized by an explicit “state” but only by its physical and biophysical properties, and so, all cells are theoretically able to undergo all events. Thus, a cell's implicit immunological “state” is inherently inferred from its properties. For example, once a T-cell expresses the CD4 receptor, it is by definition regarded as a helper T-cell, but properties dictated by this phenotype must still be explicitly defined. Configurable properties for each cell population include the amount and velocity of cells in population, the population's spatial distribution, and the composition of its membranal proteins. Per each membranal protein (e.g., receptor) population, the size of population and the typical membranal diffusion rate can be defined. Each property value in the simulation is configured using a uniform distribution (defined by minimum and maximum values) that is assigned to each cell randomly. This reflects the inherent heterogeneity of a biological system.

Each cell can be set to react in response to three types of events: (a) binding of a receptor on its surface to a neighboring membrane-bound molecule for a cell-cell interaction, (b) “sensing” the concentration of a certain molecule near a receptor, and (c) “sensing” the average concentration of a certain molecule around a cell.

Following each of these events and based on a set of rules, cellular reactions are induced: (a) secretion of molecules, (b) a change in the expression level of membranal proteins, (c) proliferation (mitosis), and (d) apoptosis. The combination of one or more events that result in one or more applied actions is referred to as a “rule.” Rules are the mechanisms with which changes in the cell's properties are introduced in the simulation.

Apart from the cells and receptors, the medium of the simulation is also specified. This medium may be a petri dish containing a substrate that sustains the cell or an organ or part of the organ that contains an extracellular medium. Several biological “regions” can be defined per each simulation (e.g., lymph node, thymus, inflammation micro-environment, etc.) and can be connected via “bridges.” Bridges allow cells to migrate across regions. In each region, different initial conditions may be specified, including the size of the region, the composition of soluble molecules in the medium, and the size of the bridges.

### Creating a simulation

**FIG. 1.**
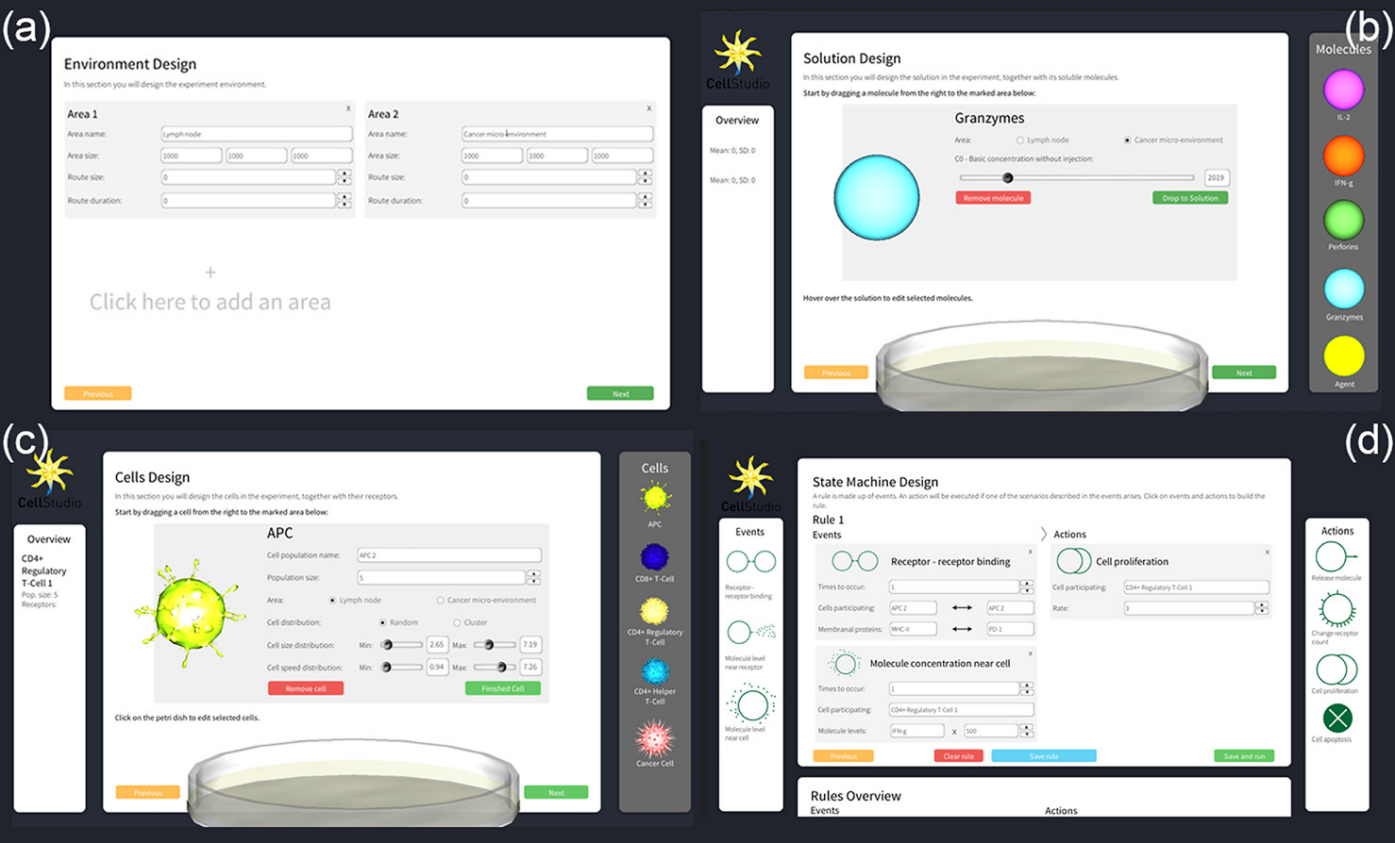
Initialization process using a GUI. (a) Design of the microenvironment of the simulation: its size, the number of compartments (referred to as “regions”), and the number of routes between regions, (b) selection of soluble molecules that inhibit the extracellular medium, (c) selection and adjustment of cell populations that take part in the simulation along with their membranal proteins, and (d) creation of the rules that define the interactions in the simulation.

Creating a new simulation in the system is done using a GUI in Unity3D. During the initialization process, the user selects and characterizes^1^ the extracellular medium (including the size of the region and the soluble molecules that inhabit it) [Figs. 1(a) and 1(b)],^2^ the cells that participate in the simulation (including the size of population, velocity, and receptor expression profiles) [Fig. 1(c)], and^3^ the rule set that dictates how cells interact with other cells and with the medium [Fig. 1(d)].

While the rules defined in the simulation (see above) represent the biological mechanism of the simulation, biophysical laws are implemented independent of the rule set and modelled entities and are calculated continuously (at significantly smaller intervals). These include the diffusion of soluble molecules in the extracellular fluid and migration of receptors along the cell surface.

### Visualization of a simulation

**FIG. 2.**
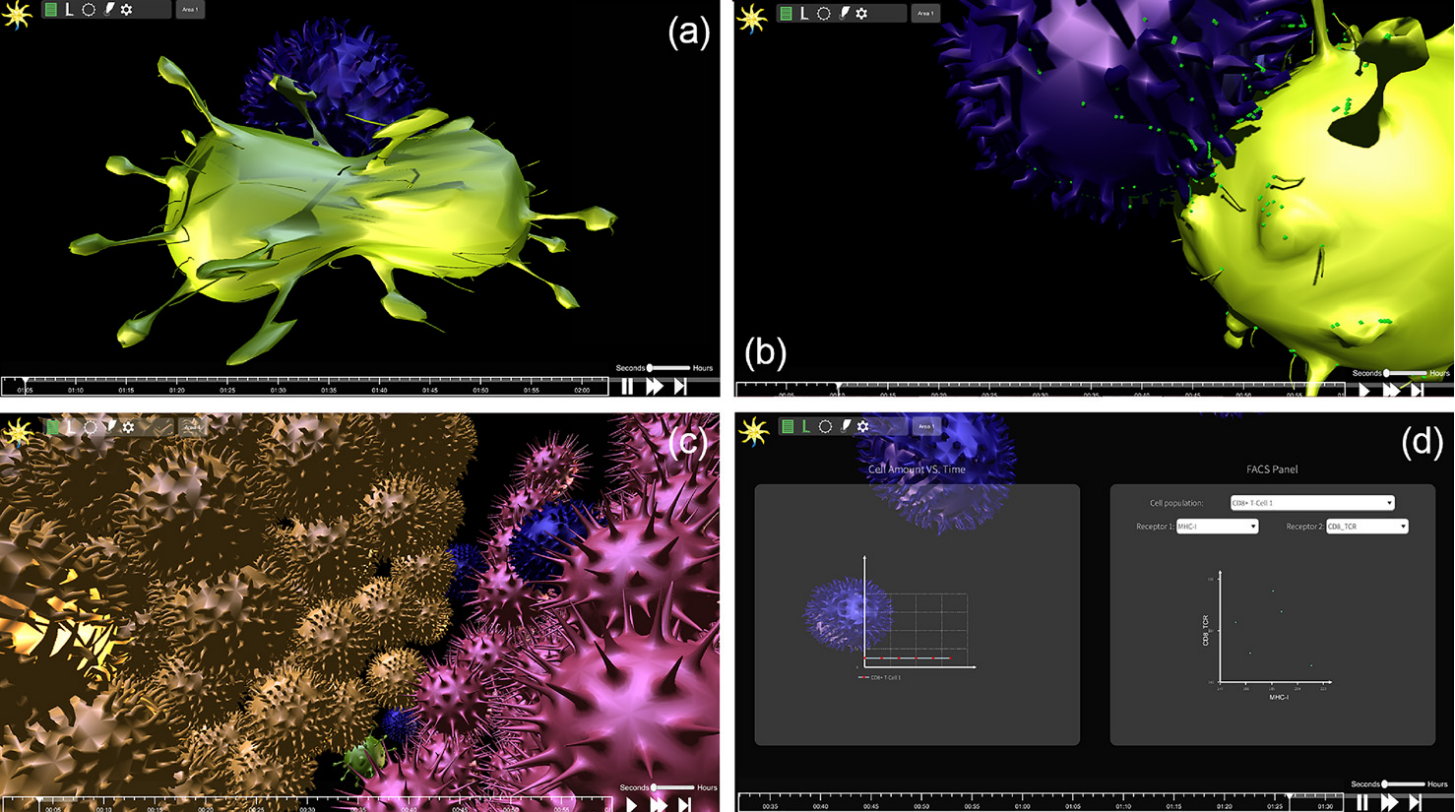
Screenshots from the simulation which depict several graphical features. (a) Cell division, (b) receptors migrating to the immune synapse in order to bind, (c) a cluster of cells which creates a tissue-like structure, and (d) graphs that display real time data from the simulation (on the left, the number of cells in each monoclonal population; on the right, a Fluorescent Activated Cell Sorting (FACS) plot).

Once the simulation is executed, the user can observe it as it is animated in real-time in 3D or via a textual output. The animated view is meant to emulate the experience of observing cellular activity under the microscope, see Fig. 2. At the default magnification, the animated objects include the cells that participate in the simulation. At higher levels of magnification, single membranal proteins are visible as they diffuse and migrate on the cell surface. The cell objects are animated in real-time, reacting to the back-end engine as it executes the simulation. Each of the cell's actions, such as death, proliferation, and “scanning” of another cell, are represented by a custom-tailored animation which was designed based on videos of cell microscopy; these animations are not movie clips prepared in advance but real-time, context-based morphing of the three-dimensional mesh which is the skeleton of the cell model. This morphing directly influences the outcome of cellular interactions, as membranal receptors' positions change as the cells morph. We are currently working on algorithms that calculate complex directional diffusion on the cell surface using the mesh, use the position of membranal receptors in the determination of the fate of cell interactions, and aim to publish the results soon.

Real-time visualization grants the user control over different aspects of the simulation. First, the user controls the timeline of the simulation: stop, play, rewind to any point in time, fast forward, and run it frame by frame. To view the simulation, a “camera”—a user-controlled window onto the simulation—is available. The user may move the camera in space, rotate, and zoom it in and out. By moving and zooming the camera at will, one can shift in real-time between a comprehensive view of the simulation with all implemented cells and a more intimate view of an immunological synapse, which is simulated according to user-defined rules and biophysical laws. Such a view may help to elucidate emergent behavior at the receptor level.

**FIG. 3.**
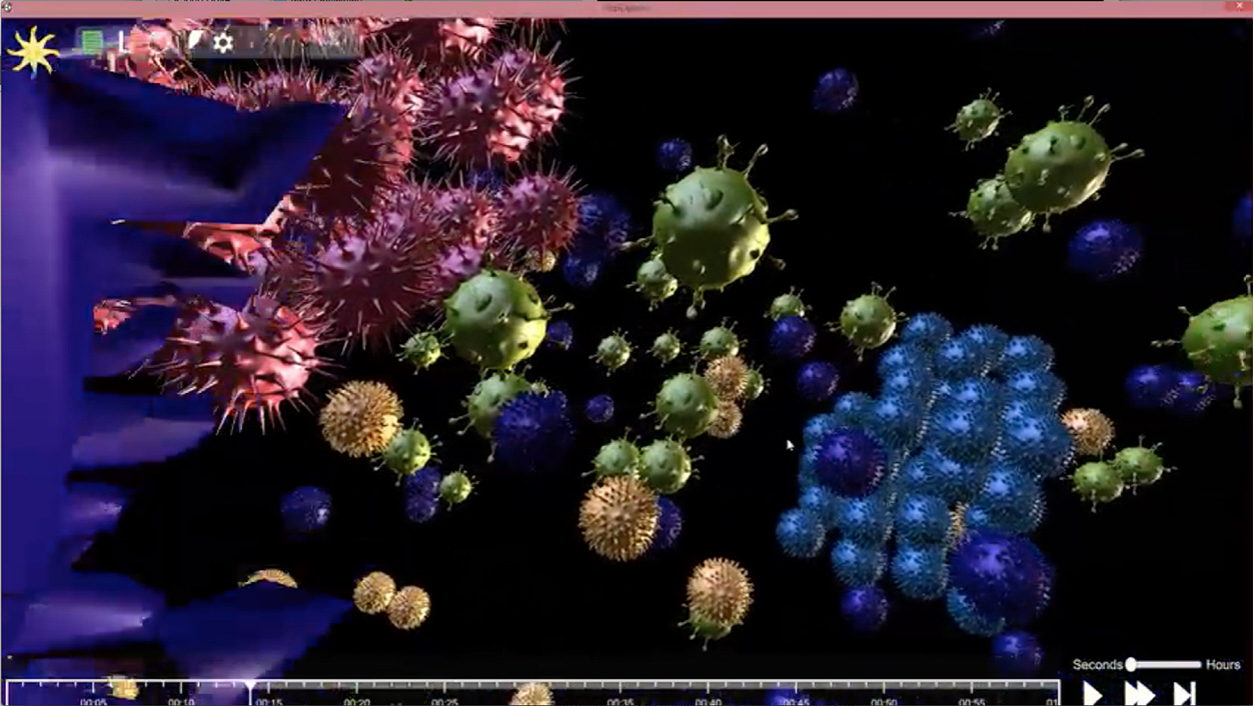
A video clip exhibiting design and execution of an experiment on Cell Studio. Featured in the order of appearance: creating a simulation, visualization of a simulation, injecting soluble molecules into the extracellular medium in mid-simulation, cell animations, diffusion of membranal proteins on the cell surface, and live plots during simulation. Multimedia view: https://doi.org/10.1063/1.5039473.1
10.1063/1.5039473.1

The user may change the course of the simulation by injecting soluble molecules at different concentrations into a region's medium. In addition, numerical data on the simulation are available in real-time by displaying: (a) FACS plots (to get receptor distribution on a monoclonal cell population) and (b) the number of cells from each monoclonal population [see Fig. 2(d)]. While the simulation is running, each event registers an on-screen alert, with an option to move the camera and examine the event up close or drop a marker on the timeline to return to it at a later time. See Fig. 3 for a move clip of the design and visualization process (Multimedia View).

It is difficult to estimate the duration the system takes to generate a simulation since it is heavily dependent upon the number of cells, membranal receptors, soluble molecules, and rules created in each simulation run. A rule of thumb would be that on a mid- to high-end computer with a modern GPU, a model containing 1000 cells each having 1000 receptors, with about 20 user-generated rules, can be simulated at a 1:1 ratio to real-time. This means that it would take the system 1 s to generate 1 s of simulation (which is also equivalent to 1 “biological” second).

A “batch mode” is available and may be used to run multiple instances of the simulation with a range of parameters. The user may select to change certain parameters across many otherwise identical simulations to learn about the effect these parameters have on the modelled system. The output of the batch mode is textual, including “snapshots” (generated in intervals of 30 s) that detail all cells (with positions), user-defined rules that were registered, and quantities from the simulation. If one of the instances of the simulation is found to be particularly interesting, it can be re-executed and visualized for in-depth inspection.

### A simple validation simulation on Cell Studio

To provide initial, high level validation, we compared trends and results from a simulation executed on Cell Studio to a corresponding simulation on an already validated system. For that, we have referred to the work by Prokopiou *et al.* on the Compucell simulation system.^54^ In this work, Prokopiou *et al.* describe a 2D simulation of an immune response to viral infection that is controlled by CD8 T-cells upon interaction with Antigen Presenting Cells (APCs). Their model included the activation of T-Cell Receptor (TCR), leading to differentiation of naïve CD8 T-cells, Soluble factors that take part in their model included the Il2 and the T-bet, a T-cell-associated transcription factor. Apoptosis of the infected cells was induced in their model by the action of caspases and by Fas-Fas ligand interactions (cell-cell contact). Their model was compared with data from C57B1/6 mice infected by intranasal H1N1 transferred with F5 cells 24 h prior to infection. CD8 responder T-Cells were assessed by flow cytometry, based on CD8, CD45.1, and CD45.2 expression. The model included the kinetics during the first days of infection (up to day 5).

In translating the above simulated experiment to terms executable on Cell Studio, we have kept as many parameters possible identical. Since the work against which we compare Cell Studio uses CompuCell in 2D, while Cell Studio simulates a 3D space, we have adapted the spatial parameters to fit our model. We have used a configuration of a single compartment, with a volume of $75\;\mu m^{3}$, simulating a lymph node. The compartment inhabits two cell populations: 3 APC cells with a radius of $10\;\mu m^{3}$ and an average speed (random movement) of 0.1 μm/min and 30 CD8 T-cells, with a radius of 1 μm and an average speed of 0.75 μm/min. Each APC cell expresses two membranal protein populations: Major Histocompatibility Complex (MHC)-I and CD80 receptors, while each CD8 T-cell expresses populations of IL2R, FasR, FasL, CD28 TCR, and CD8 TCR. Both cell populations proliferate and undergo apoptosis at rates corresponding to the above experiment. The full specification is given in the supplementary material.

The dynamics of the simulation are determined by the system of rules of events and actions. Three rules have been created, representing the interaction of a Naïve T-cell with APC which moves it through the pre-activated and activated CD8 phases, the activation of the CD8 T-cell after interaction with an APC (increase in expression of several receptors, higher chance to proliferate and die), and self-regulation of T-cells.

**FIG. 4.**
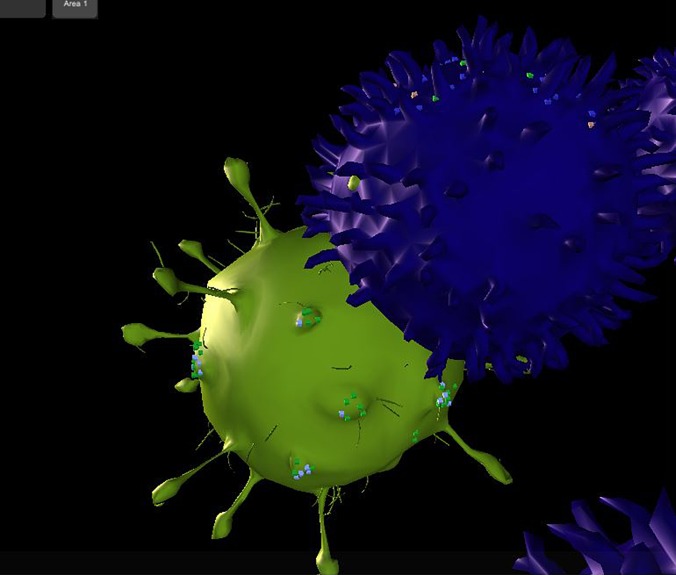
A snapshot of Cell Studio taken during a simulation run, where an activated APC (green) is in the process of antigen presentation with a Naïve T-cell (purple). The T-cell has begun forming the immune synapse with TCRs while scanning the APC (red arrow). Diffusing membranal receptors on the cell surface may be noticed: on the APC, the MHC complexes are in green, while the CD80 receptors are in light blue. The T-cell displays CD8 T-cell receptors in purple and CD28 receptors are visible in light green.

**FIG. 5.**
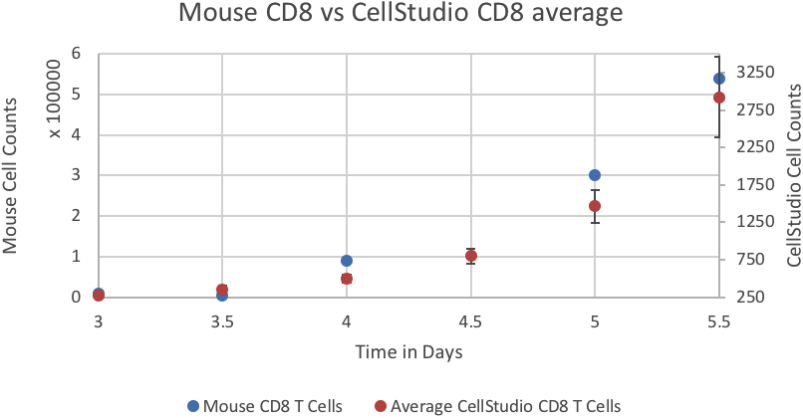
Level of all CD8 cell counts in simulation vs. the Mouse data of Prokopiou *et al.* The simulated data are the average of nine simulations, with the standard error for error bars. The number of T-cell increases with time.

Figure 4 shows a snapshot of an interaction between an APC (green) and a T-cell, taken during a real-time visualization of the simulation. A plot of results of the simulation can be viewed in Fig. 5. It may be seen that the trends and numbers are similar to those obtained by Prokopiou *et al.*

## DISCUSSION

We have developed a hybrid, cellular-level, general-purpose platform for modeling and simulation of immunological processes. The platform includes a user-friendly GUI to facilitate parametrization of a model. The model can be then simulated in two ways: either run as a single simulation in real-time, interactive 3D or as a series of simulations with a range of parameters in a non-graphical manner with a textual output (“batch mode”). The results can be plotted in real-time during the simulation. The platform includes an implementation of an extracellular medium with diffusing soluble molecules.

We have created a relatively simple validation experiment, which includes two cell populations with multiple membranal protein populations and several rules. Parameters were set according to a similar simulation created on CompuCell (itself validated against results from a “wet” experiment). We have received results which greatly resemble the results of the experiment. We treat this result as an initial “sanity check” of the simulation.

In our continuing efforts to create and validate further modules of Cell Studio, we have recently completed a module that simulates the diffusion process of membranal proteins on the cell's surface, together with recycling processes (endo- and exocytosis). As a result, we are able to implement a more biologically accurate model of juxtacrine (cell-cell) signaling. We have validated this module against a mathematical model of such processes from the literature, itself validated against *in-vitro* data (in preparation).^55^ This module (of which an in-depth description extends beyond the scope of this article) is pivotal for future simulations, while serving as another example of the way simple interactions lead to complex spatio-temporal dynamics in multi-cellular systems.

These examples of validation simulations are based on relatively simple scenarios that take into account as few parameters as possible. Due to the complexity of biological systems, these are the types of validation experiments that we believe are needed. Otherwise, the inherent uncertainty and inaccuracy in the value of parameters (most biological parameter values required for simulation are extremely hard to obtain or are unknown), specifically in an elaborate scenario, may lead to misleading results that take away from the validity of the simulation.

Enabling real-time 3D visualization in the simulation introduces two main advantages for the researcher: (a) visualization is highly important to encourage intuition and understanding of the modelled emergent behavior. We see animated visualization of a simulation as an optimal multi-dimensional representation of data; for certain types of processes, it carries more information than any other representation of data and facilitates gained insight. Of course, detailed plots of simulation results must also be displayable. (b) Interacting with a system is an instinct for researchers—intervening in the case of unintended or interesting behavior: system problems, surprising outcomes, or any irregular phenomena. The researcher may pause, rewind, or fast forward the simulation at any point, run it step by step, and control the simulation “camera.” The ability to rewind gives the simulation a “debugging” ability. Should a certain unintended event take place (all events are registered on-screen as they occur and may be marked on the timeline), the researcher may stop the simulation, inject a different molecule to the medium, and re-simulate what occurs under an alternate experimental route—this can be repeated until the desired outcome is achieved, leading to efficient online parameter optimization. We believe these two advantages—which accompany real-time 3D visualization—may aid in bridging the gap between modelers and domain experts.

The development of front-end interactive visualization requires several programmatic features: (a) development IDE—which centralizes all programming efforts, assets, and supports resource load debugging (i.e., to identify memory and CPU bottlenecks), (b) a code editor that supports a programming language and framework (this inherently includes many features like networking support, file system support, etc.), (c) graphical abilities for simulation rendering, (d) support for spatial, physics-based programming (we include in this, for example, the mesh on which the cell “skin” is laid), (e) GUI tools, which enable creation of on-screen controllers for real-time interaction during a simulation run, and (f) animation creation tools. Electing to use the Unity3D game engine as our front-end development environment has readily provided us with all these features and many more. Unity3D is also a cross-platform—deployable on almost any operation system. Beyond the natural choice of using a game engine for its available tools and benefits, it is important to note that an agent-based biological model is in many ways very similar to a computer game—it includes many actors of different properties and abilities, all of which act according to their own current state and local environment, and all together are subjected to environmental constraints.

We aimed for resource exhaustion when building the back-end algorithm of the system. For this, we utilized non-graphical GPU programming using CUDA. Since the GPU is built differently from the CPU, it is able to handle parallelization of basic tasks better. Such is, for example, the calculation of diffusion in the medium. Dividing computational load between the CPU and GPU enables better usage of both and so to enable larger simulated compartments and more simultaneous entities in the simulation. To achieve scalability for the system, we have employed a client-server architecture with constant communication between sides. The “server” can be deployed on a cluster in the “cloud” to utilize larger computational resources and reduce load from the researcher's client computer, which may not be able to handle the steeper computational requirements of the system. Moreover, cloud architecture offers load balancing which frees new resources when needed, enabling ad-hoc scaling of the back-end for specific tasks at specific times.

The inclusion of simulation of the extracellular medium—and specifically existence and diffusion of soluble factors—is important for two key processes in the simulation: non-juxtacrine cell-cell signaling and cell chemotaxis according to molecule gradients (which is currently in development). This inclusion of the constant biophysical law calculation in the simulation and combination with the biological, user-defined rules (which are calculated over longer intervals) enable many resulting phenomena to occur. For example, a given biological rule might dictate that a cell secretes a certain antibody in response to stimuli, and diffusion dictates its dispersion in the extra-cellular medium and at what concentration will this antibody reach another cell—this may, for example, yield the expected immune reaction to an invasion of a pathogen to the system.

In order to facilitate modelling of interactions occurring in multiple sites, we introduced the concept of bridges. In many physiological processes and pathologies, multiple distant tissues are involved and cells operate in more than one niche. This, however, does not imply that in all these cases an in-silico modeling of a whole organism is needed. The essence of a multi-site interaction is in many cases the migration of cells across tissues, using “bridges.” Modeling a connecting bridge is characterized by the probability of migration of a cell and some delay and bypasses the need in modeling whole organs, with enormous complexity. A bridge can describe the functional connectivity of an inflamed tissue and a lymph node, thereby allowing the migration of lymphocytes across these tissues or the connectivity to two distant tissues, allowing migration of a neoplastic cell from one localized tumor to a site of a new metastasis.

The two modes of operation (batch mode to run multiple simulations in series and animated visualization of a single run) can be integrated for optimal results: a set of simulations may be run and particularly interesting runs are observed. It should be noted that the future use of such an integrated mode would require finding ways to identify, among a large set of simulations, the ones that might be of special interest and that require further investigation.

A known problem in the field of biological modeling is the “gap” between the experimentalist (e.g., biologist), who often is not versed in programming and is not accustomed to using a computer for experimental needs, and the modeler (e.g., computer scientist), who is not the “domain expert” (see Introduction for further references). We aim to bridge this gap using a user-friendly GUI for simulation creation, built specifically to be approachable to casual users with no need for any programming knowledge. Using the above described system, of selecting cells, soluble molecules for the medium, and rules for the simulation, we expect the GUI—together with the visualized simulation—to aid in easing access to the system.

Several important challenges still remain for future development of the system. First, we plan to add an intracellular component (e.g., intracellular pathways, signaling and expression of some factor) to the simulation, by adding support to an existing intracellular modelling system, similar to Ref. 24. We aim to add support to the shared modeling format, Systems Biology Markup Language (SBML).^56^ SBML was not initially incorporated due to the added overhead in integrating it prior to the basic validation needed for this system. This is an important step in utilizing the growing library of models created by the systems biology community. We also continue to integrate further biophysical modules we see as critical for simulation of immunological scenarios, such as the above-mentioned chemotaxis.

We believe that it is important to design and build the simulation to be extensible, sharing not only complete specification of processes and models (possibly using SBML) but also single, fully specified and parameterized entities from the model, e.g., a cell population of a certain size distribution, together with their membranal proteins, biophysical properties, and even graphical representation (including mesh and texture). The use of such premade simulation assets (known as “prefabs” in the game engine jargon) may expedite the arduous work of parametrization of a simulation and help to recreate simulation conditions for comparison between researchers. It should also be noted that any vision as vast as creating a system for modeling and simulation of an immunological system must include in it the basis for a community of users, sharing knowledge and assets. This can be encouraged by creating a hub (e.g., a website or web forum—we aim to add features to our current website at www.cellstudio.info) for asset sharing.

## METHODS

### System architecture

**FIG. 6.**
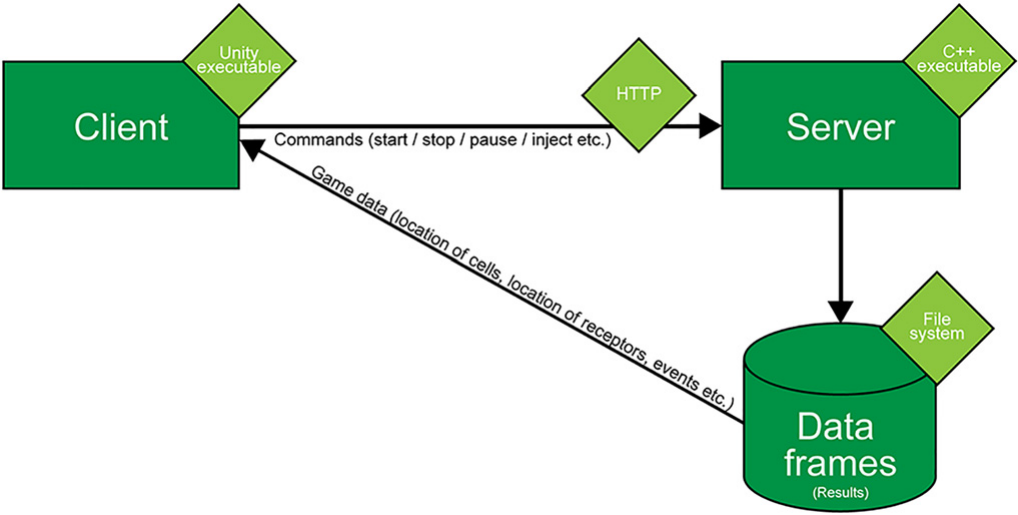
Architecture of the system. The system is separated into three modules—client, server, and file system. The server hosts the engine which runs the simulation and saves data frames, which are sent to the client, parsed, and translated into a graphical representation of the simulation.

Cell Studio is built in a client-server architecture, which is separated into three logical modules (Fig. 6): the client, the server, and the file system, which holds the data frames. The server runs the main simulation engine, constantly calculating the positions of cells and membrane proteins, diffusion of soluble molecules in the medium, chances of interaction of two cells and results of tested rules—which contain conditions posed on elements on the simulation. The output of the engine is saved in data frames, each containing a snapshot of a single time point of the simulation. Data frames are saved on the file system and sent to the client. Graphical processing is conducted on the client side, where the game engine parses data frames and displays the simulation (cells, receptors, timeline, etc.) accordingly. Control commands are sent directly to the server. This architecture can accommodate different setups: all the modules may run on a user's computer or the server and its created data frames may be hosted in the “cloud.” Using the latter architecture enables scaling of the system under heavy processing load so that more intensive calculations (e.g., more cells per simulation, more detailed cell models, more regions, etc.) may be allowed without changes to the simulation code. This architecture also removes the requirement of a high-powered computer from the end-user.

### The back end

#### General

The backend is implemented in C++ with some elements written in CUDA^51^ to be performed on the GPU for improved performance. While the CPU contains few cores optimized to handle complex calculations, the GPU contains many cores optimized to handle simple calculations. This makes it better suited to handle parallelized tasks like computation of diffusion in many grid points from many injection points and receptor movement (a simple computational action for thousands of elements). Elements written for the GPU are written to scale, using as many GPU cores as needed per calculation.

After a model is created, it is saved onto an XML file (formatted using a custom, Cell Studio specification), and maintained as a data structure in C++. Subsequently, each of the biological entities (cells, receptors, and regions) is implemented as a separate instantiated class (agent) which the simulation engine places in a single virtual 3D space. The engine also controls the time axis and handles the calculation of the logical components (rules, events, etc.).

#### Space and diffusion

An important feature of the Cell Studio framework is modeling of an extracellular medium and specifically diffusion of soluble molecules in three-dimensional space. To model the medium, we use a grid of cubes with a volume of 1 $\mu m^{3}$. Each grid cube can contain all receptors specified during the experiment initialization phase, at different concentrations. When a receptor's level in a grid cube falls beneath a value that is considered too low to generate any cellular response, it is marked and saved as $C_{\infty }$. Cells are able to react to molecule concentrations in grid cubes that they intersect with. As cells move in 3D space freely, unbound to the grid, they may intersect with several cubes at once; the level of molecules from all intersecting cubes is sampled to test for conditions of relevant user-generated rules at each time step.

For the diffusion calculation, we employ the classic Gaussian diffusion equation and continuously calculate the concentration of each molecule per each cube. When some soluble molecule concentration is injected (or secreted) into a grid cube, the simulation calculates the dispersion of the material to each adjacent cube up to a point in time and space where the concentration of the material is close enough to its value at infinity, $C_{\infty }$. The higher the molecule's diffusion value (and interchangeably, the larger the injected concentration), the larger the diffusion cloud will grow on the grid and the longer it will take it to reach $C_{\infty }$.

Cells are treated as perfect spheres. As such, the position of the cell's center together with its radius is sufficient to calculate collisions between cells. For diffusion purposes, the grid cubes that the cells occupy can also be occupied by soluble molecules, i.e., the diffusion calculation disregards the existence of cells, both in terms of soluble molecules diffusing into the volume they occupy and in terms of the drag they induce by moving in the extracellular fluid. This was necessary to enable the highly complex diffusion calculation in real-time, while still allowing modeling the effect of high concentrations of cells.

The simulation contains various user-generated rules in which a certain level of a soluble molecule near a cell is required. This mechanism effectively implements cell to cell communication for both direct and indirect interactions. The calculation of the concentration of molecules in each cube which pertains to these rules is implemented in the GPU, where the effect of the diffusion is calculated in parallel for each involved injection point (i.e., a point in the grid which is a source of soluble molecules, such as a cell secreting molecule or an external injection by pipette). The calculation is done in a “just-in-time” manner so that the molecule level will only be calculated for rule evaluation when (and if) needed.

**FIG. 7.**
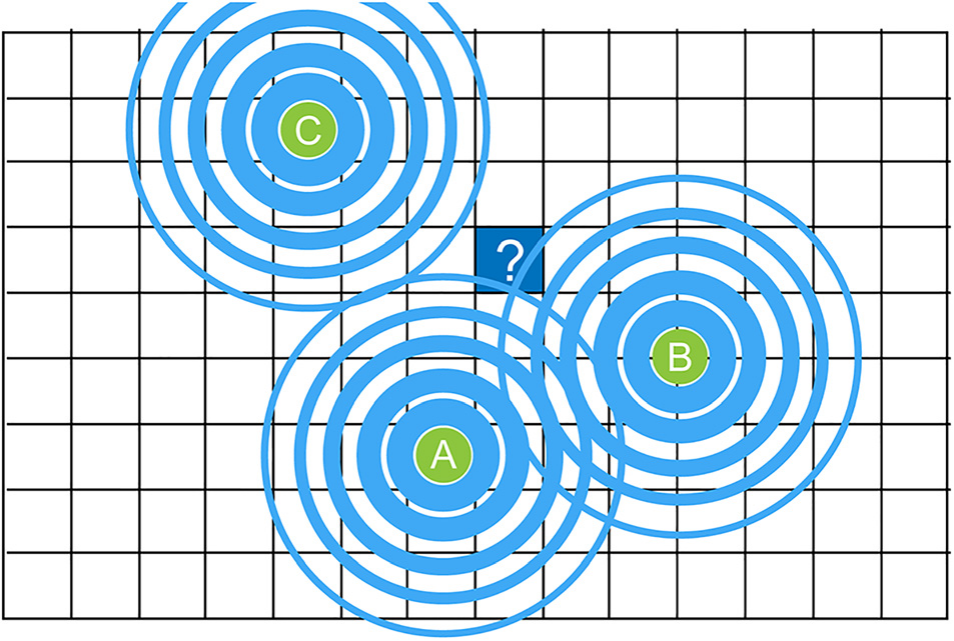
An illustration of the calculation of diffusion. To conserve computational resources, molecule levels are only calculated for the grid cube marked with a question mark if it is required for calculating the result of a rule, and the calculation will take into account only grid cubes which contain molecules that might affect the calculation of the rule. This is referred to as a “just-in-time” calculation.

Figure 7 outlines a 2D rendering (projection of a 3D original) of molecular levels in the cube marked with a question mark, in which levels will only be calculated if a rule needs to be evaluated for that grid cube at that specific point in time. Thus, if a cell which was initialized to have a rule with conditions which depend on the level of soluble molecules in the surrounding medium, upon each calculation of the rule, *local* diffusion will be calculated. The calculation is parallelized in the GPU by taking into account only “molecule secreting centers” (here A and B) which are close enough to have their molecules diffuse into the calculated area. The cumulative amount is calculated and used to evaluate the rule.

#### Membranal proteins

Receptor (and other membranal proteins) movement is currently for visualization purposes only and is calculated by moving the receptor object along the mesh of the cell object (the mesh can be thought of as “train tracks” for the receptors). Movement either occurs randomly or follows a calculated path towards a “sink point” on the object (a sink point might be, for example, an immunological synapse).

#### Cell-cell interaction

Cells may directly interact in the simulation. Successfully interaction serves as a condition for user-generated rules. Successful cell-cell interaction and signaling are determined by a condition on the number of relevant membranal proteins and the size of each cell. When two cells come close enough for a possible reaction, a linear combination of these parameters sets the probability for the reaction to occur.

### The front end

The user interface (or “front end”) of the simulation, for both the simulation parameter initialization and the visualized simulation run, is built using a game engine. We have selected to use Unity3D (Unity Technologies, San Francisco, CA, USA)^52^—currently the most widely used game engine^53^—for its graphical, scripting (using C#), network, and physics-based tools, as well as being a cross-platform (i.e., easily compiled and deployed on most computer platforms). To visualize cells, we used a set of freely available, premade cell models. A model is made up of a mesh—a three-dimensional structure—and its “skin,” the graphical texture for the mesh. Cell animations are prepared prior to the simulation run. These are not recorded animation clips but a set of directions that readjust and animate the mesh in real-time according to constraints and conditions set on the cell. These directions are created via the MegaFiers Unity package. The GUI is built using Unity3D GUI tools, which simulate a 2D canvas over the 3D simulation. Graphs are plotted using the Graph Maker Unity package.

Ethical approval for this work is not required.

## SUPPLEMENTARY MATERIAL

See supplementary material for a detailed specification for the validation simulation (see the section on A simple validation simulation on Cell Studio under Results).
